# Optimal architectures for long distance quantum communication

**DOI:** 10.1038/srep20463

**Published:** 2016-02-15

**Authors:** Sreraman Muralidharan, Linshu Li, Jungsang Kim, Norbert Lütkenhaus, Mikhail D. Lukin, Liang Jiang

**Affiliations:** 1 Department of Electrical Engineering, Yale University, New Haven, CT 06511 USA; 2 Department of Applied Physics, Yale University, New Haven, CT 06511 USA; 3 Department of Electrical and Computer Engineering, Duke University, Durham, NC 27708 USA; 4 Institute of Quantum computing, University of Waterloo, N2L 3G1 Waterloo, Canada; 5 Department of Physics, Harvard University, Cambridge, MA 02138, USA

## Abstract

Despite the tremendous progress of quantum cryptography, efficient quantum communication over long distances (≥1000 km) remains an outstanding challenge due to fiber attenuation and operation errors accumulated over the entire communication distance. Quantum repeaters (QRs), as a promising approach, can overcome both photon loss and operation errors, and hence significantly speedup the communication rate. Depending on the methods used to correct loss and operation errors, all the proposed QR schemes can be classified into three categories (generations). Here we present the first systematic comparison of three generations of quantum repeaters by evaluating the cost of both temporal and physical resources, and identify the optimized quantum repeater architecture for a given set of experimental parameters for use in quantum key distribution. Our work provides a roadmap for the experimental realizations of highly efficient quantum networks over transcontinental distances.

First developed in the 1970s, fiber-optic communication systems have boosted the rate of classical information transfer and played a major role in the advent of the information age. The possibility to encode information in quantum states using single photons and transmit them through optical channels has led to the development of quantum key distribution (QKD) systems^1^. However, errors induced by the intrinsic channel attenuation, i.e. loss errors, become a major barrier for efficient quantum communication over continental scales, due to the exponential decay of communication rate^2^. In contrast to classical communication, due to the quantum no-cloning theorem^3^, quantum states of photons cannot be amplified without any disturbance. In addition to loss errors, depolarization errors introduced by the imperfect optical channel can impair the quality of the single photon transmitted and hence the quantum information encoded.

To overcome these challenges, quantum repeaters (QRs) have been proposed for the faithful realization of long-distance quantum communication^4^. The essence of QRs is to divide the total distance of communication into shorter intermediate segments connected by QR stations, in which loss errors from fiber attenuation can be corrected. Active mechanisms are also employed at every repeater station to correct operation errors, i.e. imperfections induced by the channel, measurements and gate operations.

As illustrated in Fig. 1, loss errors can be suppressed by either heralded entanglement generation (HEG)^4^,^5^ or quantum error correction (QEC)^6^,^7^,^8^,^9^,^10^. During HEG, quantum entanglement can be generated with techniques such as two-photon interference conditioned on the click patterns of the detectors in between. Loss errors are suppressed by repeating this heralded procedure until the two adjacent stations receive the confirmation of certain successful detection patterns via *two-way* classical signaling.

Alternatively, one may encode the logical qubit into a block of physical qubits that are sent through the lossy channel and use quantum error correction to restore the logical qubit with only *one-way* signaling. Quantum error correcting codes can correct no more than 50% loss rates deterministically due to the no-cloning theorem^9^,^11^. To suppress operation errors, one may use either heralded entanglement purification (HEP)^12^,^13^ or QEC^6^,^7^,^8^,^9^,^10^ as listed in Fig. 1. In HEP, multiple low-fidelity Bell pairs are consumed to probabilistically generate a smaller number of higher-fidelity Bell pairs. Like HEG, to confirm the success of purification, *two-way* classical signaling between repeater stations for exchanging measurement results is required. Alternatively, QEC can correct operation errors using only *one-way* classical signaling, but it needs high fidelity local quantum gates.

Based on the methods adopted to suppress loss and operation errors, we can classify various QRs into three categories as shown schematically in Fig. 2, which we refer to as three generations of QRs^14^. Note that the combination of QEC for loss errors and HEG for operation errors is sub-optimum compared to the other three combinations.

Each generation of QR performs the best for a specific regime of operational parameters such as local gate speed, gate fidelity, and coupling efficiency. We consider both the temporal and physical resources consumed by the three generations of QRs and identify the most efficient architecture for different parameter regimes. The results can guide the design of efficient long distance quantum communication links that act as elementary building blocks for future quantum networks.

In this paper, we will first briefly review the chacracterstics of three generations of QRs. We use the cost coefficient as an optimization metric to compare the QR performance, and study its dependence on the individual operational parameters including coupling efficiency, gate speed and gate fidelity. Later, we present a holistic view of the optimization and illustrate the parameter regions where each generation of QRs performs more efficiently than others. Finally, we analyze the advantages and challenges of each generation of QRs and discuss the experimental candidates for their realizations.

## Results

### Three generations of quantum repeaters

The first generation of QRs uses HEG and HEP to suppress loss and operation errors, respectively^4^,^5^. This approach starts with purified high-fidelity entangled pairs with separation *L*_0_ = *L*_*tot*_/2^*n*^ created and stored in adjacent stations. At *k*-th nesting level, two entangled pairs of distance *L*_*k*−1_ = 2^*k*−1^*L*_0_ are connected to extend entanglement to distance *L*_*k*_ = 2^*k*^*L*_0_^15^. As practical gate operations and entanglement swapping inevitably cause the fidelity of entangled pairs to drop, HEP can be incorporated at each level of entanglement extension^12^,^13^. With *n* nesting levels of connection and purification, a high-fidelity entangled pair over distance *L*_*n*_ = *L*_*tot*_ can be obtained. The first generation of QRs reduces the exponential overhead in direct state transfer to only polynomial overhead, which is limited by the two-way classical signaling required by HEP between non-adjacent repeater stations. The communication rate still decreases polynomially with distance and thus becomes very slow for long distance quantum communication. The communication rate of the first generation of QRs can be boosted using temporal, spatial, and/or frequency multiplexing associated with the internal degrees of freedom for the quantum memory^5^,^16^.

The second generation of QRs uses HEG to suppress loss errors and QEC to correct operation errors^6^,^7^,^17^. First, the encoded states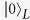 and 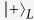 are fault-tolerantly prepared using the Calderbank-Shor-Steane (CSS) codes and stored at two adjacent stations. CSS codes are considered because of the fault tolerant implementation of preparation, measurement, and encoded CNOT gate^6^,^18^. Then, an encoded Bell pair 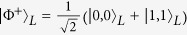 between adjacent stations can be created using teleportation-based non-local CNOT gates^19^,^20^ applied to each physical qubit in the encoded block using the entangled pairs generated through HEG process. Finally, QEC is carried out when entanglement swapping at the encoded level is performed to extend the range of entanglement. The second generation uses QEC to replace HEP and therefore avoids the time-consuming two-way classical signaling between non-adjacent stations. The communication rate is then limited by the time delay associated with two-way classical signaling between adjacent stations and local gate operations. If the probability of accumulated operation errors over all repeater stations is sufficiently small, we can simply use the second generation of QRs *without* encoding.

The third generation of QRs relies on QEC to correct both loss and operation errors^8^,^9^,^10^,^21^. The quantum information can be directly encoded in a block of physical qubits that are sent through the lossy channel. If the loss and operation errors are sufficiently small, the received physical qubits can be used to restore the whole encoding block, which is retransmitted to the next repeater station. The third generation of QRs only needs *one-way* signaling and thus can achieve very high communication rate, just like the classical repeaters only limited by local operation delay. It turns out that quantum parity codes^22^ with moderate coding blocks (200 qubits) can efficiently overcome both loss and operation errors^9^,^21^.

Note that the second and third generations of QRs can achieve communication rate much faster than the first generation over long distances, but they are technologically more demanding. For example, they require high fidelity quantum gates as QEC only works well when operation errors are below the fault tolerance threshold. The repeater spacing for the third generation of QRs is smaller compared to the first two generation of QRs because error correction can only correct a finite amount of loss errors. Moreover, quantum error correcting codes can correct only up to 50% loss error rates deterministically, which restricts the applicable parameter range for the third generation of QRs^9^.

Besides quantum key distribution, QRs can also be used for quantum state transfer. The resource requirement is mostly unchanged for the first and third generations of QRs. For the second generation, however, additional long-lived quantum memories will be required at the end (receiver) station, because the receiver has to wait for and collect all the classical signals to complete quantum teleportation.

### Comparison of three generations of QRs

To present a systematic comparison of different generations in terms of efficiency, we need to consider both temporal and physical resources. The temporal resource depends on the rate, which is limited by the time delay from the two-way classical signaling (first and second generations) and the local gate operation (second and third generations, see details below)^23^. The physical resource depends on the total number of qubits needed for HEP (first and second generations) and QEC (second and third generations)^9^,^24^. We propose to quantitatively compare the three generations of QRs using a cost function^9^ related to the required number of qubit memories to achieve a given transmission rate. Suppose a total of *N*_*tot*_ qubits are needed to generate secure keys at *R* bits/second, the cost function is defined as


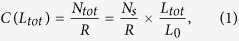


where *N*_*s*_ is the number of qubits needed per repeater station, *L*_*tot*_ the total communication distance, and *L*_0_ the spacing between neighboring stations. Since the cost function scales at least linearly with *L*_*tot*_, to demonstrate the additional overhead associated with *L*_*tot*_, the *cost coefficient* can be introduced as


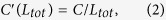


which can be interpreted as the resource overhead (qubits × time) for the creation of one secret bit over 1 km (with target distance *L*_*tot*_). Besides the fiber attenuation (with *L*_*att*_ = 20 km for telecom wavelengths), the cost coefficient also depends on other experimental parameters, in particular the coupling efficiency *η*_*c*_ (see supplementary material), the gate error probability *ε*_*G*_, and the gate time *t*_0_. For simplicity, we make the following assumptions. 1) We assume that the fidelity of physical Bell pairs 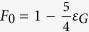 achieved with entanglement purification and measurement error probability 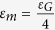 through a verification procedure (See supplementary material). 2) For third generation QRs, we restrict the search only up to 200 qubits per logical qubit considering the complexity involved in the production of larger codes and for a fair comparison with second generation of QRs. 3) We will assume that *t*_0_ is independent of code size for small encoded blocks for second and third generation QRs. We will now investigate how *C*′ varies with these parameters for three generations of QRs, and identify the optimum generation of QR depending on the technological capability.

### Coupling efficiency

**Figure 3:**
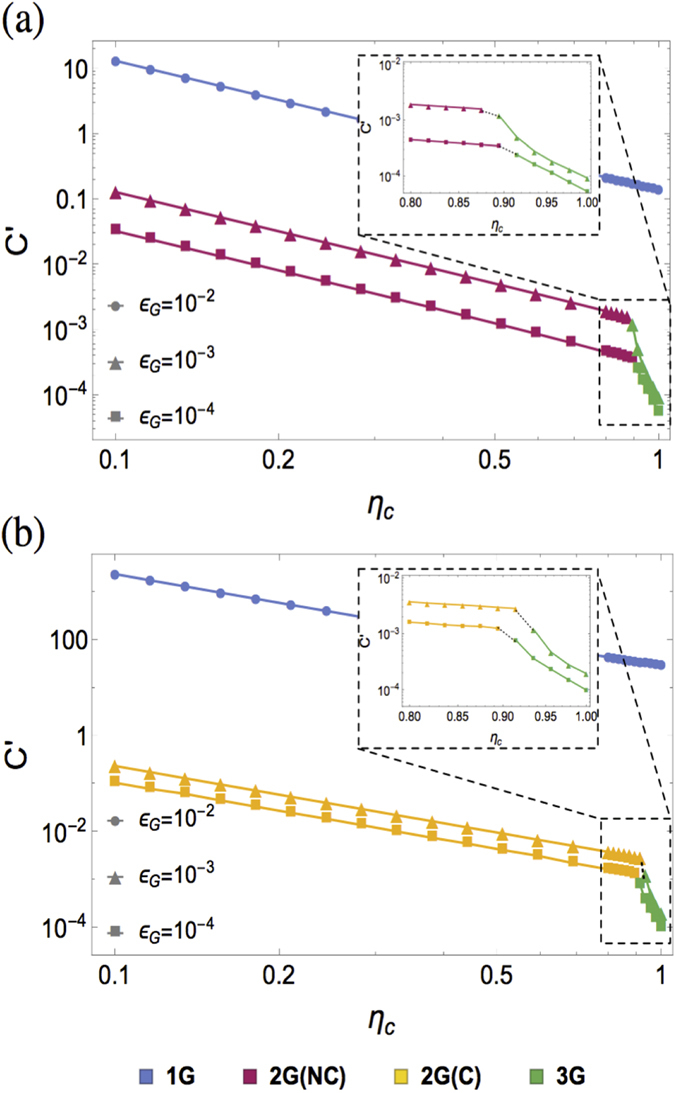
The optimized cost coefficient C’ as a function of *η*_*c*_ for *t*_0_ = 1 *μs, ε*_*G*_ ∈ {10^−2^, 10^−3^, 10^−4^}, and (**a**) *L*_*tot*_ = 1000 km, (**b**) *L*_*tot*_ = 10,000 km. The associated optimized QR protocols are indicated in different colors 2G(NC) and 2G(C) correspond to second generation without and with encoding, respectively.

The coupling efficiency *η*_*c*_ accounts for the emission of photon from the memory qubit, coupling of the photon into the optical fiber and vice versa, and the final detection of photons. The first and second generations of QRs use HEG compatible with arbitrary coupling efficiency, while the third generation relies on QEC requiring the overall transmission (including the coupling efficiency *η*_*c*_ and the channel transmission) to be at least above 50%^9^,^25^. As illustrated in Fig. 3, for high coupling efficiency (*η*_*c*_ 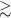 90%) the third generation of QRs has an obvious advantage over the other generations due to the elimination of two-way classical signaling. As the coupling efficiency is reduced and approaches (~90%) for quantum parity codes, the size of the coding block quickly increases and it becomes less favorable to use this approach. For coupling efficiency below ~90%, the optimization chooses the first and second generations of QRs, and then *C*′ is proportional to 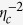 for HEG protocols heralded by two-photon detector click patterns.

If the gate error becomes large (e.g., *ε*_*G*_ = 10^−2^), the capability of correcting loss errors will be compromised for the third generation QRs. Similar trends can be observed as we fix *ε*_*G*_ and increase *L*_*tot*_. In contrast to the third generation QRs, the first and second generation QRs with HEG works well even for low coupling efficiencies.

### Speed of quantum gates

**Figure 4:**
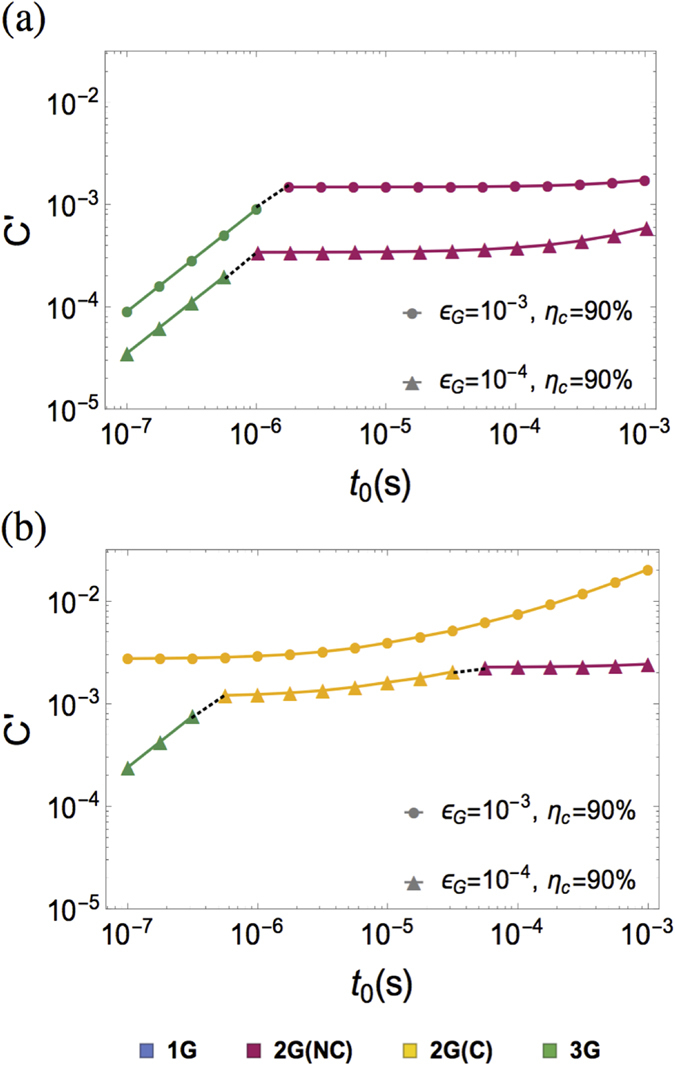
The optimized cost coefficient C’ as a function of *t*_0_ for *η*_*c*_ = 0.9, *ε*_*G*_ ∈ {10^−3^, 10^−4^}, and (**a**) *L*_*tot*._ = 1000 km, (**b**) *L*_*tot*._ = 10,000 km. The associated optimized QR protocols are indicated in different colors.

We investigate the performance of different generations of QRs for different gate times in the range 0.1 *μs* ≤ *t*_0_ ≤ 100 *μs*. As shown in Fig. 4, for high speed quantum gates (*t*_0_ 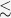 1 *μs*) the third generation of QRs provides a very fast communication rate, which makes it the most favorable protocol, with *C*′ ∝ *t*_0_. For slower quantum gates (*t*_0_ 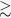 10 *μs*), the gate time becomes comparable or even larger than the delay of two-way classical signaling between adjacent stations 
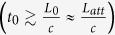
; as the third generation of QRs loses its advantage in communication rate, the second generation of QRs with less physical resources becomes the optimized QR protocol, with almost constant *C*′ for a wide range of *t*_0_.

We notice that for small gate error and intermediate distance (e.g., *ε*_*G*_ = 10^−4^ and *L*_*tot*_ = 1000 km appeared in Figs 3a and 4a), encoding might not even be necessary for the second generation of QRs, because the accumulated errors over the entire repeater network are within the tolerable range for quantum communication 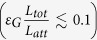. However, for larger error probability or longer distances 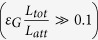, encoding is required for the second generation QRs. When *ε*_*G*_ increases from 10^−4^ to 10^−3^, the cost coefficient for the second generation of QR without encoding increases by almost a factor of 10 (Fig. 4a), while the change is less significant for the second and third generations of QRs with encoding (Fig. 4). This is because at the logical level, the change in the effective logical error probability is suppressed for the given set of parameters. The cost coefficient for the first generation of QRs 
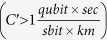
 lies beyond the scope of Fig. 4, with little dependence on *t*_0_ that is mostly negligible compared to the two-way classical signaling between non-adjacent stations 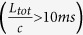.

### Gate fidelity

**Figure 5:**
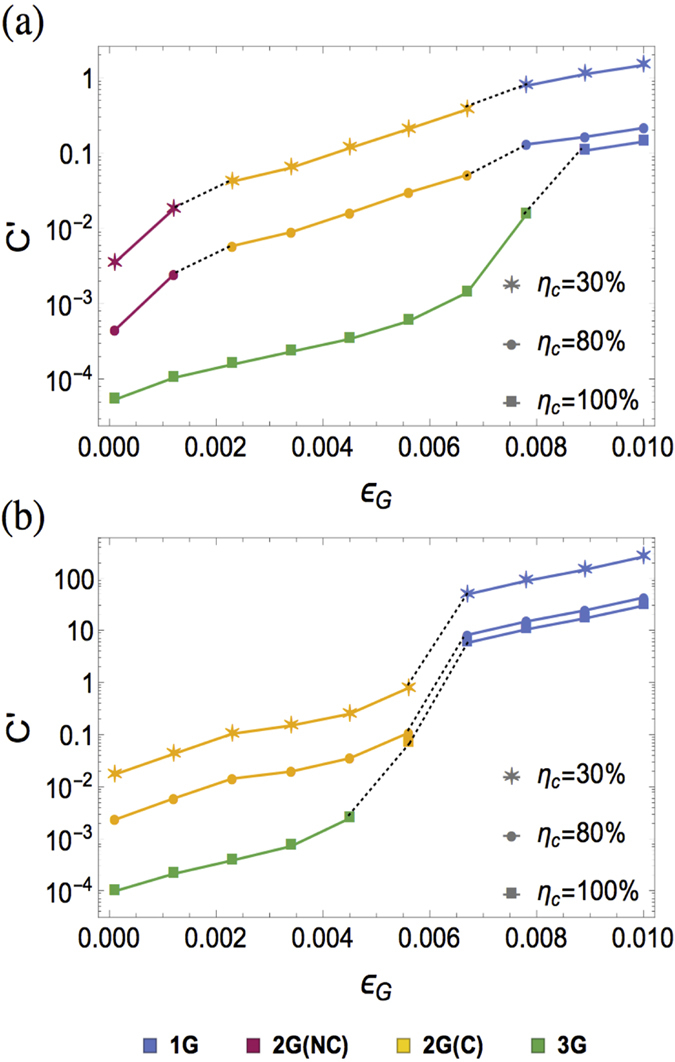
The optimized cost coefficient C′ as a function of *ε*_*G*_ for *t*_*0*_ = 1 *μs, η*_*c*_ ∈ {30%, 80%, 100%}, and (**a**) *L*_*tot*_ = 1000 km, (**b**) *L*_*tot*_ = 10,000 km. The associated optimized QR protocols are indicated in different colors.

The three generations of QRs have different thresholds in terms of gate error probability *ε*_*G*_. The first generation relies on HEP with the highest operation error threshold up to about 3%^4^. The second and third generations both use QEC to correct operation errors, with error correction thresholds of approximately 1%^26^. The gate error threshold of the second generation is slightly lower than that of the third generation, because of the extra gates required for teleportation-based non-local CNOT gates and entanglement swapping in the second generation of QRs (See supplementary material). However, since we restrict the size of the encoded block for third generation of QRs, C’ increases exponentially with *ε*_*G*_ slightly below the theoretical threshold of quantum parity codes. As illustrated in Fig. 5, for almost perfect coupling efficiency (e.g. *η*_*c*_ = 100%) and fast local operation (*t*_0_ = 1 *μs*), the third generation using QEC to correct both fiber attenuation loss and operation errors is the optimized protocol for moderate gate errors. For lower coupling efficiencies (e.g. *η*_*c*_ = 30% and 80%) with too many loss errors for the third generation to tolerate, the first and second generations with HEG yield good performance. As *ε*_*G*_ increases, there is a transition at about 0.8% (0.6%) below which the second generation is more favorable for 1000 km (10000 km).

### Optimum generation of QRs

**Figure 6:**
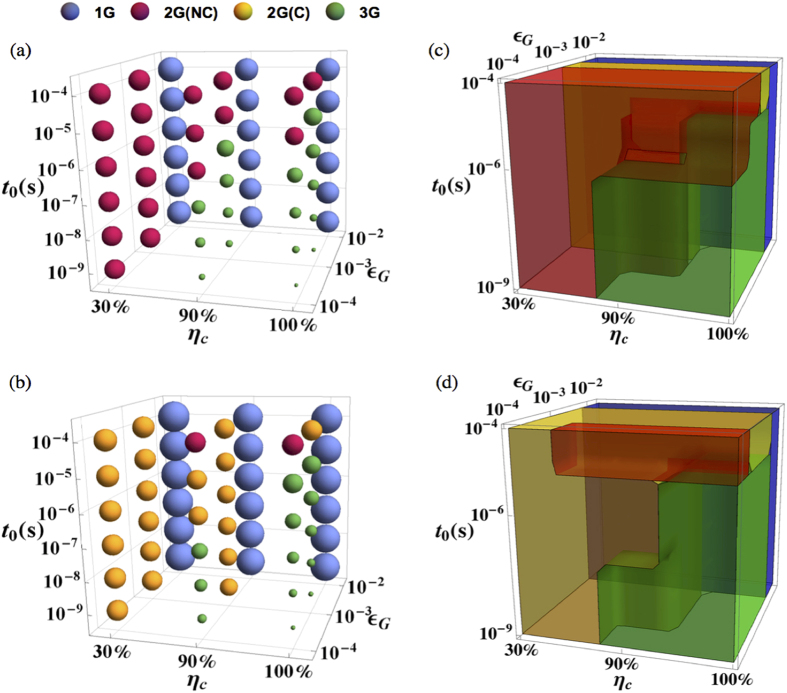
The bubble plot comparing various QR protocols in the three-dimensional parameter space spanned by *η*_*c*_, *ε*_*G*_, and *t*_0_, for (**a**) *L*_*tot*_ = 1000 km and (**b**) *L*_*tot*_ = 10,000 km. The bubble color indicates the associated optimized QR protocol, and the bubble diameter is proportional to the cost coefficient. The region plots (**c**,**d**) showing the distribution of different optimized QR protocol in the three dimensional parameter space for *L*_*tot*_ = 1000 km and *L*_*tot*_ = 10,000 km respectively. The region plot (**c**) contains a yellow region of second generation with encoding, which can be verified in a bubble plot with a finer discretization of *ε*_*G*_.

Based on the above analysis of the cost coefficient that depends on the coupling efficiency *η*_*c*_, the gate time *t*_0_, and the gate infidelity *ε*_*G*_, we may summarize the results using the bubble plot and the region plot in the three-dimensional parameter space, as shown in Fig. 6. The bubble color indicates the associated optimized QR protocol, and the bubble diameter is proportional to the cost coefficient. The parameter space can be divided into the following regions: (I) For high gate error probability 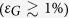, the first generation dominates; (II.A) For intermediate gate error probability, but poor coupling efficiency or slow local operation [
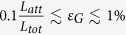
 and (*η*_*c*_ 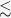 90% or *t*_0_ 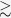 1 *μs*)], the second generation *with* encoding is more favorable; (II.B) For low gate error probability, but low coupling efficiency or slow local operation [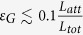 and (*η*_*c*_ 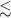 90% or *t*_0_ 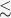 1 *μs*)], the second generation *without* encoding is more favorable; (III) For high coupling efficiency, fast local operation, and low gate error probability (*η*_*c*_ 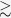 90%, *t*_0_ 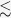 1 *μs, ε*_*G*_ 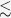 1%), the third generation becomes the most favorable scheme in terms of the cost coefficient.

## Discussion

So far, we have mostly focused on the standard procedure of HEG and HEP^4^,^5^,^12^,^13^, the CSS-type quantum error correcting codes, and the teleportation-based QEC, which all can be improved and generalized. We have also assumed the simple cost function that scales linearly with the communication time and the total number of qubits. In practice, however, the cost function may have a more complicated dependence on various resources. Nevertheless, we may extend our analysis by using more realistic cost functions to compare various QR protocols. As we bridge the architectural design of QRs and the physical implementations, we may include more variations of HEG, HEP as well as QEC and use more realistic cost functions, while the general trend and different parameter regions should remain mostly insensitive to these details. Recently, there have been growing interest in all optical quantum repeaters^14^,^27^,^28^ which does not require memory qubits. In such cases, the cost coefficient can be naturally extended to key generation rate/mode^2^ (in units bits/second/mode) and one can consider its maximization to compare different QR schemes.

The classification of QR protocols with different performance in the parameter space also provides a guideline for optimized architectural design of QRs based on technological capabilities, which are closely related to physical implementations, including atomic ensembles, trapped ions, NV centers, quantum dots, nanophotonic devices, etc. (1) The atomic ensemble can be used as quantum memory with high coupling efficiency (>80%^29^,^30^) and compatible with HEG for the first generation of QRs^31^. An important challenge for ensemble-based QRs is the use of non-deterministic quantum gates, which can be partly compensated by *multiplexing* various internal modes of the ensemble memory^5^,^16^. Alternatively, the atomic ensemble approach can be supplemented by deterministic atom-photon and atom-atom gates using Rydberg blockade, which can dramatically improve the performance of atomic ensemble approaches and make them compatible with both first and second generations of QRs^32^,^33^. (2) The trapped ions, NV centers, and quantum dots all can implement local quantum operations deterministically^34^,^35^,^36^,^37^,^38^, as well as HEG^39^,^40^,^41^,^42^. In principle, they are all compatible with the first and second generations of QRs. Although the coupling efficiency is relatively low for single emitters compared to ensembles, it can be boosted with cavity Purcell enhancement^43^ (by two orders of magnitude). With high coupling efficiency^44^,^45^, these systems can also be used for the third generation of QRs. (3) The system of nanophotonic cavity with individual trapped neutral atoms has recently demonstrated quantum optical switch controlled by a single atom with high coupling efficiency^46^,^47^, which can be used for deterministic local encoding and QEC for the third generation of QRs. Realization of similar techniques with atom-like emitters are likewise being explored. (4) The opto-electro-mechanical systems have recently demonstrated efficient coherent frequency conversion between optical and microwave photons^48^,^49^ and can potentially enable using superconducting systems^50^ for reliable fast local quantum gates for QRs.

In conclusion, we have classified various QR protocols into three generations based on different methods for suppressing loss and operation errors. Introducing the cost function to characterize both temporal and physical resources, we have systematically compared three generations of QRs for various experimental parameters, including coupling efficiency, gate time and gate fidelity. There are different parameter regions with drastically different architectural designs of quantum repeaters with different possible physical implementations. Our work will provide a guideline for the optimal design of quantum networks and help in the extension of quantum network of clocks^51^, interferometric telescopes^52^ and distributed quantum computation^20^,^53^ to global scales. In the future, the integration of different generations of QRs will enable the creation of a secure quantum internet^54^.

## Methods

### Descriptions of error models

Local two-qubit gates, e.g. CNOT gate, are characterized by the gate infidelity *ε*_*G*_. With probability 1 − *ε*_*G*_ the desired two-qubit gate is applied, while with probability *ε*_*G*_ the state of the two qubits becomes a maximally mixed state. Mathematically the imperfect two-qubit operation on qubit i and j can be expressed as


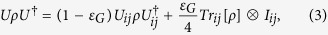


where *U*_*ij*_ stands for perfect two-qubit operation on qubit i and j, *Tr*_*ij*_[*ρ*] the partial trace over qubit i and j, and *I*_*ij*_ the identity operator for qubits i and j.

Qubit measurement error is described by the measurement infidelity ξ, which is the probability of a wrong measurement. The error models for projective measurements of states 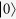 and 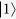 are


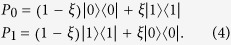


The measurement error can be suppressed by introducing an ancillary qubit for measurement and measuring both the data and the ancillary qubits. If the measurement outcomes don’t match, it can be considered as a loss error on that qubit. The contribution of the measurement error to the overall loss error is negligible given the range of the gate error rates (10^−4^–10^−2^) we are considering; if they match, then the effective measurement error is given by 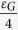
26.

In the calculations, the memory qubits are assumed to be perfect, i.e. their life-time is tremendously longer than any characteristic times involved in each scheme. In this sense the most demanding scheme is the first generation, which is optimum at long communication distances, e.g. *L*_*tot*_ = 10^4^ km, and high gate errors. The required coherence time *τ* for memory qubits is at least limited by the fundamental two-way classical communication time between Alice and Bob


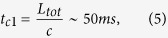


where *c* = 2 × 10^5^ km/s is the speed of light in telecom-wavelength optical fiber. Recent experiments with trapped ions, superconducting qubits, solid state spins and neutral atoms have demonstrated quantum memory life-times approaching or exceeding this characteristic value. For the second generation, the characteristic communication time is


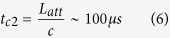


where *L*_*att*_ = 20 km at telecomm wavelength. The corresponding coherence times are far less demanding than that of the first generation, which relieves the strong life-time requirements on memory qubits and makes the two generations more plausible in practice. Note that when the operation time *t*_0_ becomes comparable or larger than the characteristic communication time, it is then the operation time *t*_0_ that puts limits to the coherence time *τ*. Third generation QRs are not limited by the two way communication time because it is a fully one way communication scheme.

### Entanglement fidelity and entanglement purification

The density matrix of an imperfect Bell pair can be expressed in the Bell basis as the following


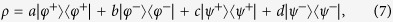


where 
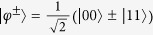
 and 
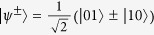
 are the four Bell states. The fidelity of the pair is thus defined as


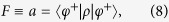


Both the first and second generations of QRs rely on generating elementary entangled pairs between neighboring repeater stations and then extending the entanglement to longer distances. Practically, however, an entangled pair generated between neighboring stations may not be a perfect Bell pair. In addition, for the first generation, HEP is needed at higher levels of entanglement extension to overcome operation errors. With the technique of HEP, the fidelity of entangled pairs can be boosted to near-unity at the cost of reducing the total number of them and purified pairs can be connected to obtain longer entangled pairs or used as resources for implementing remote quantum gates.

We note that with imperfect quantum operations and measurements, there is an upper bound on the fidelity of entangled pairs even with entanglement purification. It is in general, a function of the density matrix of raw Bell pairs *ρ*, gate infidelity *ε*_*G*_ and measurement infidelity *ξ*, and depends on the specific purification protocol one uses. Using Deutsch *et al.* purification protocol (see the supplementary for details), the value of this upper bound can be approximated as


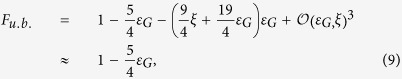


in which we assume depolarized states for input raw Bell pairs. This approximate expression holds at small *ε*_*G*_’s (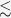
1%). In our calculations and comparison, the temporal resources and physical resources consumed in obtaining purified pairs between neighboring stations are not accounted; elementary entangled pairs generated between neighboring stations are assumed to directly take this asymptotic value. The associated additional cost in the purification can be easily added as an overhead into the cost function.

For HEP at higher levels in the first generation, we consider two widely used entanglement purification protocols: Deutsch *et al.* protocol^12^ and Dür^13^ *et al.* protocol. Compared to other purification schemes, the Deutsch protocol reaches higher fidelities with fewer rounds of purification so its upper bound is used as the fidelity of elementary pairs between neighboring stations. The Dür *et al.* purification protocol is very similar to the Deutsch *et al.* protocol, except that one of the two pairs, called auxiliary pair, is never discarded and will be prepared in the same state in each round of purification. This is sometimes also call “entanglement pumping”. The Dür *et al.* purification protocol saves qubit resources by keeping making use of the auxiliary pair, while the state preparation in each purification round is costly in time as a trade-off and if one round fails, the whole purification needs to be started over again. Details of the two protocols can be found in the supplementary. Note that, in general, despite differences in experimental requirements and efficiencies, the choice of purification protocols will not affect the big picture of scheme optimization.

## Additional Information

**How to cite this article**: Muralidharan, S. *et al.* Optimal architectures for long distance quantum communication. *Sci. Rep.* **6**, 20463; doi: 10.1038/srep20463 (2016).

## Supplementary Material

Supplementary Information

## Figures and Tables

**Figure 1:**
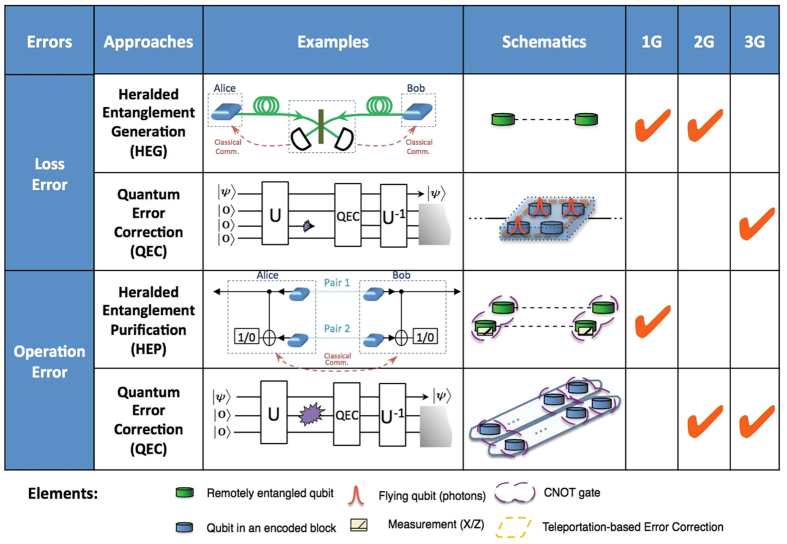
A list of methods to correct loss and operation errors. Depending on the methods used to correct the errors, QRs are categorized into three generations.

**Figure 2:**
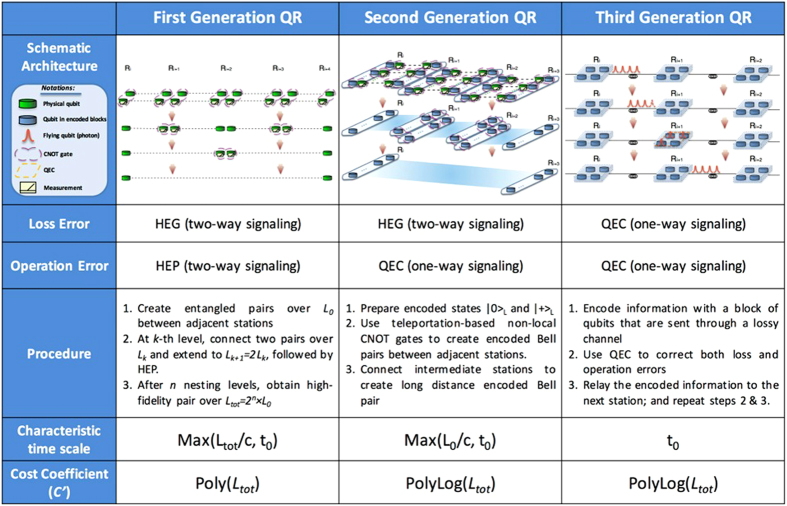
Comparison of three generations of QRs.
