# Supplementary material for: Optimal architectures for long distance quantum communication
Source: Sci Rep. 2016 Feb 15;6:20463. doi: 10.1038/srep20463 (PMC4753438; doi:10.1038/srep20463)
Supplement: Supplementary Information [file srep20463-s1.pdf]

Supplemental Material:
Optimal architectures for long distance quantum communication

Sreraman Muralidharan[1*], Linshu Li[2*], Jungsang Kim[3],
Norbert Lütkenhaus[4], Mikhail D. Lukin[5], and Liang Jiang[2*]
[1]*Department of Electrical Engineering, Yale University, New Haven, CT 06511 USA*
[2]*Department of Applied Physics, Yale University, New Haven, CT 06511 USA*
[3]*Department of Electrical and Computer Engineering, Duke University, Durham, NC 27708 USA*
[4]*Institute of Quantum computing, University of Waterloo, N2L 3G1 Waterloo, Canada and*
[5]*Department of Physics, Harvard University, Cambridge, MA 02138, USA*
(Dated: December 4, 2015)

I. QUANTUM ENTANGLEMENT GENERATION, PURIFICATION AND CONNECTION

A. Heralded entanglement generation with two-photon detection

Using two photon-detection in the middle[1, 2], the success probability of one trial of generating entanglement between two memory qubits in neighboring stations is

$$p = \frac{1}{2}\eta_c^2 e^{-L_0/L_{att}}, \tag{I.1}$$

where L_0 is the spacing between neighboring stations and η_c is the coupling efficiency accounting for the emission of the photon from the memory qubit, "upload" of the photon into the optical fiber, "download" of the photon from the fiber and the final detection of photons.

B. Deutsch *et. al.* and Dür *et. al.* purification protocols

We study the purification with two input pairs characterized by density matrices ρ_1 and ρ_2 ($\rho_1 = \rho_2$ in the case of Deutsch *et. al.* protocol). As mentioned in the maintext, we express the density matrices in the Bell basis $\{|\varphi^+\rangle, |\varphi^-\rangle, |\psi^+\rangle, |\psi^-\rangle\}$. With input states$\{a_1, b_1, c_1, d_1\}$ and $\{a_2, b_2, c_2, d_2\}$, in the presence of gate infidelity ϵ_G and measurement infidelity ξ, the success probability P and the purified state characterized by the diagonal elements $\{a, b, c, d\}$ are the

---

*Equal contribution

following

$$\begin{aligned}
P &= (1-\epsilon_G)^2\{[\xi^2+(1-\xi)^2][(a_1+d_1)(a_2+d_2)+(b_1+c_1)(c_2+b_2)]+2\xi(1-\xi)[(a_1+d_1)(b_2+c_2)+ \\
&\quad (b_1+c_1)(a_2+d_2)]\}+\frac{1}{2}[1-(1-\epsilon_G)^2] \\
a &= \frac{1}{P}\{(1-\epsilon_G)^2[(\xi^2+(1-\xi)^2](a_1a_2+d_1d_2)+2\xi(1-\xi)(a_1c_2+d_1b_2)\}+\frac{1}{8}[1-(1-\epsilon_G)^2)] \\
b &= \frac{1}{P}\{(1-\epsilon_G)^2[(\xi^2+(1-\xi)^2](a_1d_2+d_1a_2)+2\xi(1-\xi)(a_1b_2+d_1c_2)\}+\frac{1}{8}[1-(1-\epsilon_G)^2)] \\
c &= \frac{1}{P}\{(1-\epsilon_G)^2[(\xi^2+(1-\xi)^2](b_1b_2+c_1c_2)+2\xi(1-\xi)(b_1d_2+c_1a_2)\}+\frac{1}{8}[1-(1-\epsilon_G)^2)] \\
d &= \frac{1}{P}\{(1-\epsilon_G)^2[(\xi^2+(1-\xi)^2](b_1c_2+c_1b_2)+2\xi(1-\xi)(b_1a_2+c_1d_2)\}+\frac{1}{8}[1-(1-\epsilon_G)^2)]
\end{aligned} \tag{I.2}$$

C. Entanglement Swapping

Entanglement swapping is used in the first generation and second generation without encoding to extend the distance of entanglement. With imperfect CNOT operation and measurements, the diagonal elements $\{a, b, c, d\}$ in the Bell basis of the resulting state obtained from connecting deterministically the input pairs $\{a_1, b_1, c_1, d_1\}$ and $\{a_2, b_2, c_2, d_2\}$ is

$$\begin{aligned}
a &= (1-\epsilon_G)\{(1-\xi)^2(a_1a_2+b_1b_2+c_1c_2+d_1d_2)+\xi(1-\xi)[(a_1+d_1)(b_2+c_2)+(b_1+c_1)(a_2+d_2)] \\
&\quad +\xi^2(a_1d_2+d_1a_2+b_1c_2+c_1b_2)\}+\frac{\epsilon_G}{4} \\
b &= (1-\epsilon_G)\{(1-\xi)^2(a_1b_2+b_1a_2+c_1d_2+d_1c_2)+\xi(1-\xi)[(a_1+d_1)(a_2+d_2)+(b_1+c_1)(b_2+c_2)] \\
&\quad +\xi^2(a_1c_2+c_1a_2+b_1d_2+d_1b_2)\}+\frac{\epsilon_G}{4} \\
c &= (1-\epsilon_G)\{(1-\xi)^2(a_1c_2+c_1a_2+b_1d_2+d_1b_2)+\xi(1-\xi)[(a_1+d_1)(a_2+d_2)+(b_1+c_1)(b_2+c_2)] \\
&\quad +\xi^2(a_1b_2+b_1a_2+c_1d_2+d_1c_2)\}+\frac{\epsilon_G}{4} \\
d &= (1-\epsilon_G)\{(1-\xi)^2(a_1d_2+d_1a_2+c_1b_2+b_1c_2)+\xi(1-\xi)[(a_1+d_1)(b_2+c_2)+(b_1+c_1)(a_2+d_2)] \\
&\quad +\xi^2(a_1a_2+b_1b_2+c_1c_2+d_1d_2)\}+\frac{\epsilon_G}{4}
\end{aligned} \tag{I.3}$$

Note that deterministic entanglement swapping is crucial for long distance quantum communication using QRs. Otherwise the success probability of entangling two qubits separated by L_{tot} drops exponentially as L_{tot} increases.

II. IMPLEMENTATION AND OPTIMIZATION

A. First generation

The first generation of QRs corrects photon loss and operation errors with HEG and HEP, respectively. To overcome the exponential decay in key generation rate induced by photon loss, the total distance L_{tot} is divided into 2^n segments (n is called nesting level[1, 5]) and elementary entangled

pairs are generated within each segment, i.e. over repeater spacing $L_0 = \frac{L_{tot}}{2^n}$. An entangled pair covering the total distance L_{tot} can be generated via n levels of entanglement swapping: at each level, two adjacent entangled pairs are connected so that an entangled pair over twice the distance is produced.

However, entanglement swapping necessarily reduces the fidelity of the entangled pairs due to the following two reasons: 1) entanglement swapping involves CNOT operation and measurements, which themselves are imperfect in reality and will introduce noise. 2) Despite imperfect operations, the connection of two imperfect Bell pairs gives a pair with lower fidelity. So multiple rounds of entanglement purification may need to be incorporated at each level to maintain the fidelity, so that the final pair covering L_{tot} is sufficiently robust for secure key distribution.

In the optimization, to determine the best scheme from the first generation QRs, we first fix

- Total distance L_{tot},
- Coupling efficiency η_c
- Gate error rate ϵ_G , and thus the fidelity of elementary Bell pairs $F_0 = 1 - \frac{5}{4}\epsilon_G$
- Gate time t_0

and we vary the following parameters:

- Number of nesting level: N
- Number of rounds of purification at each level: $\overrightarrow{M} = (M_0, M_1, M_2, \cdots, M_N)$
- Choice of entanglement purification protocol: Deutsch or Dür

In carrying out the time resource consumed in generating one remote pair, we adopted similar approximations and derivations as in a previous work[1], with three major changes:

1. We allow arbitrary number of rounds of purification at each level in the optimization, and hence schemes selected could potentially be better optimized.
2. Without losing generality, we use four-state protocol, instead of six-state protocol, in calculating the secure fraction at the asymptotic limit.
3. For long-distance quantum communication, we only consider *deterministic* entanglement swapping and take into account the gate operation time t_0.

For a given set of N and $\overrightarrow{M}$, detailed derivations of expressions of temporal and physical resource consumed in Deutsch and Dür are given below.

1. *Deutsch et al. entanglement purification protocol*

The temporal resource to generate one bit of raw key, T_{Deu}, can be calculated as follows:

$$T_{Deu} = T_0 \cdot \{ (\frac{3}{2})^N (\prod_{x=0}^{N-1} A_{Deu}[\text{N-x}])(\frac{1}{P_0} A_{Deu}[0] + B_{Deu}[0]) + \sum_{y=1}^{N} (\frac{3}{2})^{N-y} B_{Deu}[y] \prod_{x=0}^{N-(y+1)} A_{Deu}[\text{N-x}] + \frac{t_0}{T_0} \sum_{y=1}^{N} (\frac{3}{2})^{N-y} \prod_{x=0}^{N-y} A_{Deu}[\text{N-x}] \}, \quad \text{(II.1)}$$

where

$$A_{Deu}[i] \equiv (\frac{3}{2})^{M_i} \prod_{x=0}^{M_i-1} \frac{1}{P_{Deu}(M_i-x,i)}$$
$$B_{Deu}[i] \equiv (\frac{t_0}{T_0}+2^i) \sum_{y=0}^{M_i-1} (\frac{3}{2})^y \prod_{x=0}^{y} \frac{1}{P_{Deu}(M_i-x,i)}. \quad \text{(II.2)}$$

Here, $A_{Deu}[i]$ accounts for the time consumed in the preparation of Bell pairs for purification, and $B_{Deu}[i]$ includes gate operation time and the *two-way* classical signaling time associated with confirming the success of purification. $P_{Deu}(i,j)$ is the success probability of the ith-round of purification at the jth nesting level with Deustch purification protocol. $T_0 = \frac{L_0}{c}$ is the time unit for the *two-way* classical signaling between neighboring stations, where $c = 2 \times 10^5 km/s$ in optical fiber. The secure key generation rate, R_{secure} (sbit/s), can be written as

$$R^{Deu}_{secure} = r_{secure} \cdot \frac{1}{T_{Deu}}, \quad \text{(II.3)}$$

where r_{secure} is the asymptotic secure fraction and in the four-state protocol can be approximately expressed as

$$r_{secure} = Max[1-2h(Q),0], \quad \text{(II.4)}$$

where $Q = \frac{Q_X+Q_Z}{2}$ is the average quantum bit error rate (QBER) and $h(Q) = -Q\log_2 Q - (1-Q)\log_2(1-Q)$ is the binary entropy function. $Q_{X/Z}$ can be calculated from the density matrix of the entangled shared by Alice and Bob in the end. The physical resource, in terms of the number of memory qubits, consumed at half a station can be written as

$$Z_{Deu} = 2^{\sum_{i=0}^{N+1} M_i}, \quad \text{(II.5)}$$

and the cost function becomes

$$C = \frac{2^{N+1} \cdot Z_{Deu}}{R^{Deu}_{secure}}. \quad \text{(II.6)}$$

2. Dür et al. entanglement purification protocol

The temporal resource to generate one bit of raw key, T_{Dr}, can be calculated as follows:

$$T_{Dr} = T_0 \cdot \{ (\frac{3}{2})^N (\frac{1}{P_0} A_{Dr}[0] + B_{Dr}[0]) (\prod_{x=0}^{N-1} A_{D\ddot{u}r}[\text{N-x}]) + \sum_{y=1}^{N} (\frac{3}{2})^{N-y} B_{Dr}[y] \prod_{x=0}^{N-(y+1)} A_{D\ddot{u}r}[\text{N-x}]$$
$$+ \frac{t_0}{T_0} \sum_{y=1}^{N} (\frac{3}{2})^{N-y} \prod_{x=0}^{N-y} A_{D\ddot{u}r}[\text{N-x}] \}, \quad \text{(II.7)}$$

where

$$A_{Dr}[i] \equiv \prod_{x=0}^{M_i-1} \frac{1}{P_{Dr}(M_i-x,i)} + \sum_{y=0}^{M_i-1}\prod_{x=0}^{y} \frac{1}{P_{Dr}(M_i-x,i)}$$

$$B_{Dr}[i] \equiv (\frac{t_0}{T_0}+2^i) \sum_{y=0}^{M_i-1}\prod_{x=0}^{y} \frac{1}{P_{Dr}(M_i-x,i)}. \quad \text{(II.8)}$$

Here, $A_{Dr}[i]$ accounts for the time consumed in the preparation of Bell pairs for purification (notice the extra term due to entanglement pumping), and $B_{Dr}[i]$ includes gate operation time and the *two-way* classical signaling time associated with confirming the success of purification. $P_{Dr}(i,j)$ is the success probability of the ith-round of purification at the jth nesting level with Dür purification protocol. Because of the unique entanglement pumping mechanism, the physical resource consumed at half a station is reduced compared to Deustch purification protocol and expressed as

$$Z_{\text{Dï¿œr}} = N + 2 - |\{M_i : M_i = 0\}|. \quad \text{(II.9)}$$

The derivations of secure key generation rate and hence cost function are similar to those in the previous section.

B. Second generation without encoding

The second generation of QRs relies on generating encoded Bell pairs between neighboring stations and performing error correction during entanglement swapping at the encoded level. With encoding and error correction, physical gate error rates and imperfections in raw Bell pairs are suppressed to higher orders and thus entanglement can be extended to very long distances with high fidelity. However, if we are interested in the best schemes at low gate error rate $\epsilon \lesssim 10^{-3}$ and total distance $L_{tot} \sim 10^3 km$, the encoding may turn out unnecessary and resources can be saved by simply generating elementary pairs between neighboring stations and implementing entanglement swapping. Fixing the same parameters as the previous section, we vary the following parameters

- Number of memory qubits per half station: N
- Spacing between neighboring stations: L_0
- Number of rounds of elementary entanglement generation trial: $n_{E.G.}$

The secure key generation rate, R_{secure} (sbit/s), can be written as

$$R_{secure} = \frac{[1-Prob(0,n_{E.G.})]^{\lceil \frac{L_{tot}}{L_0} \rceil} \cdot r_{secure}}{n_{E.G.} \cdot (T_0+t_0)}, \quad \text{(II.10)}$$

where basic communication time $T_0 = \frac{L_0}{c}$ and r_{secure} follows the same definition above. $Prob(i,n_0) = \begin{pmatrix} M \\ n_0 \end{pmatrix} p^{n_0}(1-p)^{M-n_0}$ is the probability to generate i elementary pairs with M qubits in the two half nodes after n_0 rounds of entanglement generation. Therefore, $1-Prob(0,n_{E.G.})$ means the probability to have *at least* one entangled pair between two neighboring stations. Note

that frequency and spatial multiplexing may needed to be incorporated during the transmission of flying qubits and entanglement swapping, respectively. The cost function that will be optimized can be written as

$$C = \frac{2M \cdot \lceil \frac{L_{tot}}{L_0} \rceil}{R_{secure}}. \tag{II.11}$$

C. Second generation with encoding

For second generation QRs with encoding, encoded Bell pairs are created between neighboring repeater stations and later an encoded entanglement swapping operation is performed at every QR station to generate an encoded Bell pair between distant stations. As in the case of first generation QRs, Bell pairs are generated using HEG between neighboring stations with a high fidelity. These Bell pairs are used as a resource to perform teleportation based CNOT gates between neighboring stations, thereby realizing an encoded CNOT operation between neighboring QR stations. The depolarization error on the data qubits can be modeled as

$$\rho' = \mathcal{E}(\rho) = (1-\epsilon_d)\,\rho + \frac{\epsilon_d}{4}\sum_{k=0}^{3} \sigma_k \rho \sigma_k. \tag{II.12}$$

The probability of an error being detected in any one (X or Z) of the measurements is given by,

$$\epsilon_{X/Z} = \epsilon_d + \epsilon_G + 2\xi + \frac{2}{3}(1-F_0) + O(\epsilon_G, \xi)^2 \tag{II.13}$$

Any $[[N, k, 2t+1]]$ CSS code can correct up to t X-errors and t Z-errors respectively. Taking this into account, the probability of correctly and incorrectly decoding the qubit are given by,

$$p^{2G}_{correct(X/Z)} = \sum_{k=0}^{t} \binom{N}{k} \epsilon^k_{X/Z}(1-\epsilon_{X/Z})^{(N-k)}, \tag{II.14}$$

$$p^{2G}_{incorrect(X/Z)} = \sum_{k=t+1}^{N} \binom{N}{k} \epsilon^k_{X/Z}(1-\epsilon_{X/Z})^{(N-k)} \tag{II.15}$$

respectively. Accounting for logical errors in odd number of repeater stations, quantum bit error rates for X and Z basis after R repeater stations is given by,

$$Q_{(X/Z)} = \frac{1}{2}\left[1 - \left(p^{2G}_{correct(X/Z)} - p^{2G}_{incorrect(X/Z)}\right)^R\right]. \tag{II.16}$$

Where the effective quantum bit error rate is given by$Q = \frac{1}{2}(Q_X + Q_Z)$. The success probability of the protocol is conditioned on having enough Bell pairs between neighboring stations to apply a teleportation based CNOT gate. We can then obtain the key generation rates similar to the case of second generation without encoding. We consider the Steane [[7,1,3]] code, Golay [[23,1,7]] code and the QR [[103,1,19]] codes in our optimization.

D. Third generation QRs

Third generation QRs rely on encoded qubits to relay data from one repeater station to the next where an error correction operation is performed. Since there is just one round of upload and download between the memory qubit and the fiber for third generation QRs, the probability that the photon reaches the neighboring station is given by $\eta_c e^{-L_0/L_{att}}$. Unlike second generation QRs with encoding, teleportation based error correction (TEC) is performed within every repeater station locally for third generation QRs. Teleportation based error correction requires an encoded CNOT gate between the incoming encoded qubit block (with loss and operation errors) and encoded qubit block (with no loss errors) at every repeater station. R is the incoming encoded block and S is the encoded block from the encoded Bell pair. The depolarization errors on blocks R and S can be modeled as,

$$\rho_R' = \mathcal{E}_R\left(\rho_{RS}\right) = \eta\left(1-\epsilon_d\right)\rho_R + \frac{\eta\epsilon_d}{4}\sum_{k=0}^{3}\sigma_k\rho\sigma_k + \left(1-\eta\right)|vac\rangle\langle vac| \tag{II.17}$$

$$\rho_S' = \mathcal{E}_S\left(\rho_{RS}\right) = \left(1-\epsilon_d\right)\rho_S + \frac{\epsilon_d}{4}\sum_{k=0}^{3}\sigma_k\rho\sigma_k, \tag{II.18}$$

0 To be consistent with[7], we make the following assumptions in our analysis. 1) Errors are not propagated between repeater stations. 2) Each qubit has an independent error. The effective X/Z error detected at the measurement (Y errors are detected in both X and Z measurements) is given by,

$$\epsilon_{X/Z} = (\epsilon_d + \frac{\epsilon_G}{2} + \xi)\eta + O(\epsilon_G,,\xi)^2. \tag{II.19}$$

We consider (n,m) quantum parity codes given by

$$|\pm\rangle_L = \frac{1}{2^{n/2}}(|0\rangle^{\otimes m} \pm |1\rangle^{\otimes m})^{\otimes n}, \tag{II.20}$$

in our analyses. The outcome of the measurement of the logical operators X_L and Z_L for TEC can be determined through a majority voting procedure discussed in detail in [7]. There are three possible outcomes of the majority voting procedure: a) Heralded failure leading to the inability to perform a majority voting with probability $p^{3G}_{unknown(X/Z)}$. b) Perform a majority voting and correctly decoding the qubit with probability $p^{3G}_{correct(X/Z)}$. c) Perform a majority voting and incorrectly decoding the qubit with probability $p^{3G}_{incorrect(X/Z)}$. Here, we treat the three events as independent for the measurement of the logical operators for simplicity. The success probability accounting for no heralded failure in any one of the R repeater stations is given by,

$$P_{succ} = (1 - p^{3G}_{unknown(X/Z)})^R \tag{II.21}$$

Accounting for errors in odd number of repeater stations, the quantum bit error rate for X/Z bases is given by,

$$Q_{(X/Z)} = \frac{1}{2}\left[1 - \left(\frac{p^{3G}_{correct(X/Z)} - p^{3G}_{incorrect(X/Z)}}{p^{3G}_{correct(X/Z)} + p^{3G}_{incorrect(X/Z)}}\right)^{R}\right] \tag{II.22}$$

The asymptotic secure key generation rates is given by,

$$R_{secure} = Max[\frac{P_{succ}}{t_0}\{1 - 2h(Q)\}, 0], \tag{II.23}$$

where t_0 is the time taken to apply local operations. The cost function for the (n, m) quantum parity codes is given by

$$C = \frac{2m \cdot n\lceil \frac{L_{tot}}{L_0} \rceil}{R_{secure}}. \tag{II.24}$$

For a fair comparison with second generation QRs, where a largest code of $[[103, 1, 19]]$ code was used, we restrict the maximum qubits for the third generation quantum repeaters to be 200. We restrict the search of (n, m) quantum parity codes within the range $2 \leq (m, n) \leq 20$.

---

[1] Sangouard, N., Dubessy, R. & Simon, C. Quantum repeaters based on single trapped ions. *Phys. Rev. A* **79,** 042340 (2009)

[2] Sangouard, N., Simon, C., de Riedmatten, H. & Gisin, N. Quantum repeaters based on atomic ensembles and linear optics. *Rev. Mod. Phys.* **83**, 33–80 (2011).

[3] Deutsch, D. *et al.* Quantum privacy amplification and the security of quantum cryptography over noisy channels. *Phys. Rev. Lett.* **77,** 2818 (1996)

[4] Briegel, H-J., Dür, W., Cirac, J. I. & Zoller, P. Quantum Repeaters: The Role of Imperfect Local Operations in Quantum Communication. *Phys. Rev. Lett.* **81,** 5932 (1998).

[5] Dür, W., Briegel, H-J., Cirac, J. I. & Zoller, P. Quantum repeaters based on entanglement purification. *Phys. Rev. A* **59,** 169 (1999).

[6] Bratzik, S., Abruzzo, S., Kampermann, H. & Bruï¿œ, D. Quantum repeaters and quantum key distribution: The impact of entanglement distillation on the secret key rate. *Phys. Rev. A* **87,** 062335 (2013)

[7] Muralidharan, S., Kim, J., Lï¿œtkenhaus, N., Lukin, M. & Jiang, L. Ultrafast and Fault-Tolerant Quantum Communication across Long Distances. *Phys. Rev. Lett.* **112,** 250501 (2014)
